# High Dose Antipsychotic Therapy Prescription Amongst Patients Admitted to the Psychiatric Intensive Care Unit (PICU), Mill View Hospital, Hove

**DOI:** 10.1192/bjo.2024.533

**Published:** 2024-08-01

**Authors:** Dorcas Awolaran, Samina Monir

**Affiliations:** Sussex Partnership NHS Foundation Trust, West Sussex, United Kingdom

## Abstract

**Aims:**

Acutely ill patients on PICU are likely to be on High dose antipsychotic treatment (HDAT), which poses a risk to their physical health. Current guidelines require that appropriate criteria be met before prescription, and close physical health monitoring after prescription of HDAT. This audit aims to assess practices regarding prescription of HDAT to patients on PICU, Mill View Hospital, Hove according to standard guidelines.

**Methods:**

Ten of the 38 patients admitted to PICU at Mill View Hospital between January and June 2023 were on HDAT and thus were eligible for this audit. The revised prescribing observatory for mental health topic 1h 3e audit tool was used to collect data regarding the patients. Data was collected from the clinical records including electronic, paper notes and uploaded drug charts and forms.

**Results:**

The age range was between 21–56 with an average age of 35. Eight of the 10 patients were white British, 2 were of another ethnic group or ethnicity unknown. All the 10 patients had clinical reasons for HDAT prescription clearly documented at the start of the treatment which ranged from cross titration of antipsychotics to treatment resistance to standard treatments.

Of the 10 patients on HDAT, 6 of them had documented clinical review in the 3 months, 1 had documented clinical review in last 3 to 6 months, 3 had no clearly documented review of clinical response in the last year.

Only 7 out of 10 had their temperature, pulse, blood pressure, body mass index and electrocardiogram (ECG) clearly documented. Seven of the 10 patients had their full blood count, urea and electrolytes, liver function tests, blood glucose, plasma lipid tests done and clearly documented in last year. Eight of the 10 patients had their serum prolactin checked while none of the patients had their creatinine phosphokinase checked and clearly documented in the last year. Only 2 of the patients had clearly documented examination of extrapyramidal side effects (EPSE) in the last one year.

**Conclusion:**

This audit demonstrates that although clinical reasons for HDAT prescription were documented for all patients, current standard guidelines for HDAT prescription regarding regular review and physical health monitoring were either not being met, or not clearly documented.

